# Interaction of cCMP with the cGK, cAK and MAPK Kinases in Murine Tissues

**DOI:** 10.1371/journal.pone.0126057

**Published:** 2015-05-15

**Authors:** Stefanie Wolfertstetter, Jörg Reinders, Frank Schwede, Peter Ruth, Elisabeth Schinner, Jens Schlossmann

**Affiliations:** 1 Department of Pharmacology and Toxicology, Institute of Pharmacy, University of Regensburg, Regensburg, Germany; 2 Institute of Functional Genomics, University of Regensburg, Regensburg, Germany; 3 Biolog Life Science Institute, Bremen, Germany; 4 Department of Pharmacology, Toxicology and Clinical Pharmacy, Institute of Pharmacy, University of Tübingen, Tübingen, Germany; Griffith University, AUSTRALIA

## Abstract

cAMP and cGMP are well established second messengers that are essential for numerous (patho)physiological processes. These purine cyclic nucleotides activate cAK and cGK, respectively. Recently, the existence of cCMP was described, and a possible function for this cyclic nucleotide was investigated. It was postulated that cCMP plays a role as a second messenger. However, the functions regulated by cCMP are mostly unknown. To elucidate probable functions, cCMP-binding and -activated proteins were identified using different methods. We investigated the effect of cCMP on purified cyclic nucleotide-dependent protein kinases and lung and jejunum tissues of wild type (WT), cGKI-knockout (cGKI KO) and cGKII-knockout (cGKII KO) mice. The catalytic activity of protein kinases was measured by a (γ-^32^P) ATP kinase assay. Cyclic nucleotide-dependent protein kinases (cAK, cGKI and cGKII) in WT tissue lysates were stimulated by cCMP. In contrast, there was no stimulation of phosphorylation in KO tissue lysates. Competitive binding assays identified cAK, cGKI, and cGKII as cCMP-binding proteins. An interaction between cCMP/MAPK and a protein-protein complex of MAPK/cGK were detected via cCMP affinity chromatography and co-immunoprecipitation, respectively. These complexes were abolished or reduced in jejunum tissues from cGKI KO or cGKII KO mice. In contrast, these complexes were observed in the lung tissues from WT, cGKI KO and cGKII KO mice. Moreover, cCMP was also able to stimulate the phosphorylation of MAPK. These results suggest that MAPK signaling is regulated by cGMP-dependent protein kinases upon activation by cCMP. Based on these results, we propose that additional cCMP-dependent protein kinases that are capable of modulating MAPK signaling could exist. Hence, cCMP could potentially act as a second messenger in the cAK/cGK and MAPK signaling pathways and play an important role in physiological processes of the jejunum and lung.

## Introduction

The principle of cyclic nucleotides as intracellular signaling molecules is well established in regard to the purinergic cyclic nucleotides cyclic adenosine monophosphate (cAMP) and cyclic guanosine monophosphate (cGMP). However, more recent data has suggested that the pyrimidinic cyclic nucleotides cyclic cytidine monophosphate (cCMP) and cyclic uridine monophosphate (cUMP) might also exert an intracellular function. These cyclic nucleotides can be synthesized in eukaryotic cells by soluble nucleotidyl cyclases, such as soluble guanylyl cyclase (sGC) or soluble adenylyl cyclase (sAC) [1,2]. Furthermore, the synthesis of pyrimidinic cyclic nucleotides has been detected in bacterial cells (e.g., Pseudomonas aeruginosa) and was shown to be stimulated by bacterial toxins (e.g., edema factor) [3–5]. These observations raise the question of whether possible target proteins and/or effector systems of these cyclic nucleotides exist. Therefore, studies have sought to elucidate a possible role for these cyclic nucleotides as intracellular second messengers [6]. cCMP activates purified cGMP-dependent protein kinase I (cGKI) isoforms cGKIα and cGKIβ, cGMP-dependent protein kinase II (cGKII) and cAMP-dependent protein kinase (cAK) [7,8]. Additionally, exogenous application of cCMP induced cGK-mediated modulation of vascular relaxation, and the inhibition of platelet aggregation via cGMP-kinase I was detected using transgenic mice [7]. The binding of cCMP to cAKRIα (a regulatory subunit of cAK) was observed in mammalian cells [9]. However, to date it is not known whether cCMP can function as an intracellular messenger molecule *in vivo*. A critical point is whether the concentration of cCMP *in vivo* is sufficient to activate the identified signaling proteins. Moreover, a selective signaling pathway for cCMP has not yet been demonstrated. Although cCMP could act via pathways that are usually activated by other cyclic nucleotides such as cAMP/cGMP, further downstream effectors may exist. Moreover, the stimuli inducing cCMP synthesis could differ from those of the other cyclic nucleotide systems, and therefore a specific role for cCMP could be elucidated. Hence, the discovery of further cCMP targets is important for the identification of probable cCMP functions. To elucidate possible targets of cCMP in tissues, we performed cCMP kinase activity assays and cCMP-affinity chromatography of lung or intestinal murine tissues and analyzed the relevant proteins using mass spectrometry and immunoblotting analysis. These tissues were selected because cGKI and/or cGKII are present in high concentrations. cGKI was originally purified from lung (which contains high amounts of cGKI) [10,11], intestinal tissues including jejunum contain high concentrations of cGKI and cGKII [11,12]. We confirmed that cGKI and cAK are cCMP-interacting proteins, and identified cGKII and MAPK as possible cCMP signaling molecules.

## Materials and Methods

### Materials

All cyclic nucleotides (3',5'-cCMP (cCMP); N^4^, 2'- O- Dibutyrylcytidine- 3', 5'- cyclic monophosphate (DB-cCMP); 3’,5’-cAMP (cAMP); 3’,5’-cGMP (cGMP)), the cCMP or cGMP coupled agarose beads and the corresponding EtOH-NH-agarose were provided by the BIOLOG Life Science Institute (Bremen, Germany). The different primary antibodies were purchased as indicated in the different sections. PhosStop tablets were ordered from Roche Diagnostics (Basel, Switzerland). The ProteoSilver Silver Stain Kit and all the other chemicals were purchased from Sigma-Aldrich (St. Louis, MO, USA).

### Animals

129/Sv- cGKII-knockout (cGKII KO) and 129/Sv- wild type (WT) mice of both genders were used at an age >8 weeks. 129/Sv- cGKI-knockout (cGKI KO) mice were used at an age of 4–6 weeks due to the low survival rate of the adult animals. We kindly thank Prof. Franz Hofmann for providing breeding pairs of the cGKI and cGKII KO mice. All mice were bred and maintained in the animal facilities of the University of Regensburg. Surgery was performed under anesthesia (2% isofluran). All experiments conformed to the guidelines for the care and use of laboratory animals published by the US National Institute of Health and were approved by the local governmental committee.

### Tissue lysate preparation

After anesthetizing and perfusing the mice through the abdominal aorta with 0.9% NaCl-solution containing 10 IU/mL heparin, the organs were removed and cleaned. The tissues were immediately frozen in liquid nitrogen and stored at -80°C before use. The homogenization was performed with a tissue homogenizer (Ultraturrax, Janke&Kunkel KG, IKA-Wert, Staufen i. Br., Germany) in extraction buffer (20 mM Tris-HCl, 250 mM NaCl, and 2% Lubrol [v/v] [nonaethyleneglykol nonadodecylether], pH 8.0) containing protease inhibitors (1 μM leupeptin, 300 μM PMSF, and 1 mM benzamidine). Additionally, a phosphatase inhibitor cocktail (PhosStop) was added in the phosphorylation experiments to conserve the phosphorylation state of the proteins. The homogenates were centrifuged (10 min, 4°C, 13,000xg), and the supernatant fractions were separated for further experiments and the determination of the protein concentrations using the Qubit system (Life Technologies, Darmstadt, Germany).

### Measurement of kinase activity

The catalytic activity of protein kinases, which was dependent on the concentration of cyclic nucleotides, was measured using a (γ-^32^P) ATP kinase assay. Recombinant protein kinases cGKI (both isoforms, bovine) and cGKII (mouse) were purified in our lab following expression in SF9 cells as described previously [13–15]. Purified protein kinase cAK was purchased from Sigma-Aldrich. The tissue lysates were prepared as described above except that a different extraction buffer was used (20 mM Tris-HCl, 100 mM NaCl, pH 8.0, and protease inhibitors). The reaction was started by adding 20 μL of the protein sample to the reaction mixture (50 mM MES, pH 6.9, 0.4 mM EGTA, 1 mM Mg-acetate, 10 mM NaCl, 0.1% (w/v) BSA, 10 mM DTT, 40 μM substrate peptide IRAGtide (Sequence: RRRVSVAV) or VASPtide (Sequence: RRKVSKQE), 2 μM cAK-inhibitor peptide, 0.1 mM [γ-^32^P] ATP (100 cpm/pmol) and ± cNMP at increasing concentrations). The K_a_ values for the different cyclic nucleotides were determined with GraphPad Prism based on the results of three to four experiments, using increasing concentrations of cNMP (1 nM to 50 mM). K_a_ values in experiments with tissue lysates were determined in the same way as in the experiments with purified enzyme, but only two representative concentrations were shown. To eliminate cAK-phosphorylation, a cAK-inhibitor peptide (AS_5-24_) was applied in all assays with tissues. This substance is a potent, synthetic peptide inhibitor of cAMP-dependent protein kinase with a K_i_ value of 2.3 nM [16], but is also a weak inhibitor of cGMP-dependent protein kinase. However, the conditions were selected using a concentration of AS_5-24_ were cAK is completely inactivated and cGK is inhibited to a minimal amount [17]. After incubation (5 min at 30°C), the samples were transferred to Whatman P-81 filter papers (Sigma-Aldrich) and the reaction was stopped by the addition of 75 mM H_3_PO_4_. Rotiscint scintillation liquid (Carl Roth GmbH & Co. KG, Karlsruhe, Germany) was added to the dried phosphocellulose papers to measure the counts per minute using a β-counter (Tri Carb 2800TR Liquid Scintillation Analyzer, Perkin Elmer, Rodgau, Germany).

### Competition binding assay

cCMP-binding proteins were identified using competitive binding assays modified for the analysis of tissues from [9]. Tissue lysates were mixed with additional extraction buffer (see above) and centrifuged (15 min, 4°C, 24,000xg) to separate non-lysed proteins and cell fractions. Then, we added N^4^-(6-aminohexyl)-cCMP agarose (4-AH-cCMP) beads (ligand density ~ 6 μmol / mL of settled gel) and corresponding control agarose (EtOH-NH, Ctrl.) beads. The mixtures were blocked with 3% BSA and washed three times with extraction buffer. Clarified protein lysate (700 μg), 100 μM isobutyl-methylxanthine and 1 mM DTT were added to the beads. Next, 200 μM, 2 mM cCMP or 2 mM cAMP was added (indicated with + cNMP) or omitted (indicated with—cNMP). For some experiments 8-(2-aminoethylthio)-3’,5’-cGMP agarose (ligand density ~6.5 μmol/mL of settled gel)was used and for competition 200 μM cGMP was added. Untreated tissue lysate (1 μg/μL, indicated with input) and purified enzyme (1.5 ng/μL) were used as the controls. After the incubation (4°C, overnight), the samples were centrifuged (3 min, 4°C, 20,000xg) and the sedimented beads were washed another three times. Afterwards, the washing solution was completely removed and the proteins were eluted with Laemmli buffer. The bound proteins were analyzed by silver staining and immunoblotting.

### Co-immunoprecipitation

Co-immunoprecipitation was used to detect cCMP-dependent protein complexes. Protein A-Sepharose beads (Sigma-Aldrich, antibody-binding capacity 6 mg per mL) were blocked with 3% BSA, washed three times with extraction buffer (see above) and incubated with a specific primary MAPK antibody (p44/42 MAPK, Cell Signaling Technology, Leiden, The Netherlands) at 4°C for 4 hours. Then, the beads were washed again and the clarified protein lysate (700 μg per IP experiment) was added prior to incubation (+ 1 mM DTT, 4°C, overnight). The subsequent steps were identical to those described above (competition binding assay).

### Phosphorylation analysis

Tissue lysates (50 μg) were added to buffer (50 mM MES, 10 mM ATP, pH 7.2, protease and phosphatase inhibitors) for the phosphorylation studies and treated with DB-cCMP, (100 μM, BIOLOG) for the indicated timepoints (15/30/60 min, 37°C). As a control, unstimulated tissue lysate (50 μg, indicated with `us`) was used. Control samples (us) were treated the same as the cyclic nucleotide stimulated samples except that water was added instead of DB-cCMP. Data were expressed as x-fold MAPK phosphorylation relative to the untreated control samples. Quantification of MAPK phosphorylation was always in comparison to total MAPK and the unstimulated sample. To analyze the role of cAK in MAPK phosphorylation, a cAK-inhibitor peptide (AS_5-24_, 10 μM) was added or omitted. AS_5-24_ was only added in lung or jejunum WT samples, in every single sample (‘us’, 15, 30, 60) together with the buffer without preincubation (indicated with + AS_5-24_). The phosphorylation state of the stimulated and unstimulated protein samples (each 2.5 μg/μL) was detected by SDS-PAGE and Western blotting.

### SDS-PAGE and Western blotting

The samples were pretreated as described in the sections above. Proteins were separated by 11.5% sodium-dodecyl sulfate-polyacrylamide gel electrophoresis (SDS-PAGE; equal volumes (15 μL, concentration as indicated in figure legends) of protein were loaded in every lane) and transferred to a PVDF membrane using a semi-dry transfer system (also described in [18]). For one hour, the blotted membrane was blocked with 5% nonfat milk in TBST (10 mM Tris-HCl, 150 mM NaCl, pH 8.0, with 0.1% Tween 20), and then washed with TBST. For protein detection, the blot was incubated with a primary antibody (4°C, overnight) and then with a corresponding goat anti-rabbit IgG secondary antibody coupled to horseradish peroxidase (dilution 1:50,000, room temperature, 2 hours) (Dianova GmbH, Hamburg, Germany). The rabbit primary antibodies used were: rabbit anti-cGKIcommon (polyclonal, dilution 1:200, detection of both isoforms), rabbit anti-cGKIα (polyclonal, dilution 1:500) [11], rabbit anti-cGKII (polyclonal antibody, dilution 1:1000) [19], rabbit anti-cAKRIα/β (1:500, polyclonal) (Santa Cruz Biotechnology, Heidelberg, Germany), polyclonal rabbit anti-p44/42 MAPK and monoclonal rabbit anti-p-p44/42 MAPK (dilution 1:1000 for both) (Cell Signaling Technology). As control untreated jejunum or lung tissue lysate (1 μg/μL, indicated with input) was used. In some cases, the membranes had to be stripped for further detection; after washing three times with TBST, a stripping solution (100 mM NaOH, 2% SDS, 0.5% DTT, 37°C, 30 min) was used. Then, retreatment with another antibody was possible. The proteins were visualized with Clarity Western ECL substrate using the ChemiDoc MP system (both Bio-Rad Laboratories GmbH). The quantification was performed with ImageLab Software (Bio-Rad Laboratories GmbH).

### Silver staining and mass spectrometric identification

In addition to SDS-PAGE and Western blotting, cCMP-binding proteins were visualized by silver staining using the ProteoSilver Plus Silver Stain Kit (Sigma-Aldrich) as described in the manufacturer’s instructions. Bands of interest were excised and washed according to the protocol of Shevchenko et al. [20]. Briefly, gel bands were washed twice in an alternating fashion with 50 μL 50 mM NH_4_HCO_3_ (washing buffer A) and 25 mM NH_4_HCO_3_ in 50% acetonitrile (washing buffer B). The reduction of disulfide bridges was accomplished by incubation in 10 mM DTT in washing buffer A at 56°C for 30 min. Cysteines were carbamidomethylated by incubation with 5 mM iodoacetamide in washing buffer A for 30 min. Subsequently, the washing steps were repeated, and the gel pieces were dried in a vacuum centrifuge. They were rehydrated using 6 μL of trypsin solution (10 ng/μL in washing buffer A) and incubated overnight at 37°C for in-gel digestion. The resulting peptides were eluted with 20 μL of 5% formic acid and subjected to nano-LC-MS/MS-analysis on an Ultimate 3000 nano-HPLC-system (Dionex GmbH, Idstein, Germany) using a 1 h binary gradient directly coupled to a QTOF mass spectrometer (QStar XL, Applied Biosystems, Darmstadt, Germany) as previously described [21]. The tandem-MS spectra were aligned with the Uniprot-database using the Protein Pilot 4.5 software (ABSciex, Darmstadt, Germany) applying the two-peptide rule.

### Statistical Analysis

All data are expressed as the mean ± SEM. Error bars in the Figures indicate SEM. The t-test was used to calculate significant differences between two means; the ANOVA test was used to assess differences between three or more means. P-values are indicated as follows: * p<0.05, ** p<0.01, ***p<0.001; n.s. not statistically significant (p>0.5); n describes the number of experiments.

## Results

### Activation of cyclic nucleotide dependent kinases by cCMP in vitro and in tissues

**Fig 1:**
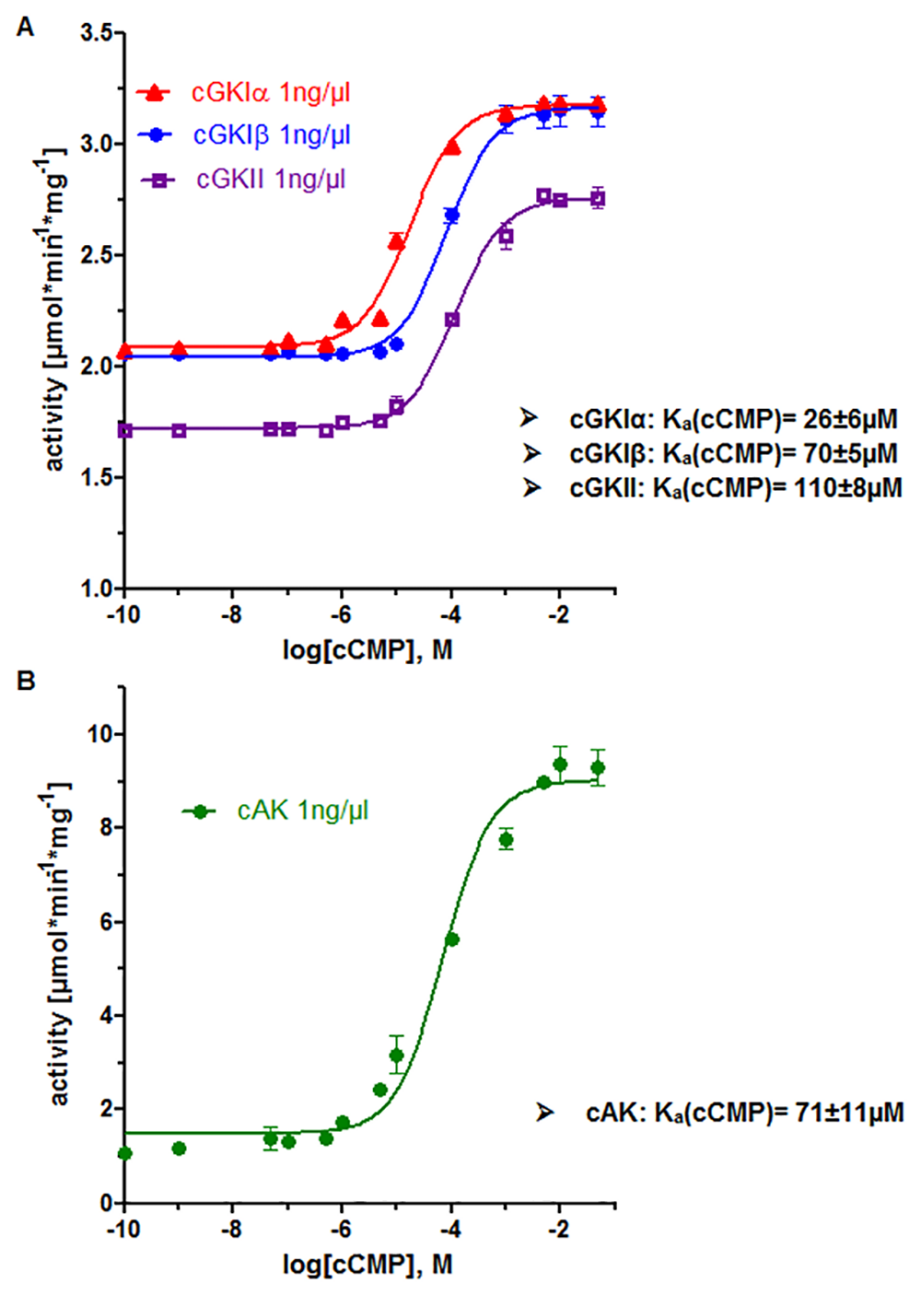
Activation of cGKIα, cGKIβ and cAK by cCMP (A) *In vitro* concentration response curves of purified, recombinant bovine cGKI isozymes and murine cGKII with cCMP as an activator and IRAGtide (RRRVSVAV) as a substrate (B) *In vitro* concentration response curves of purified, recombinant bovine cAK with cCMP and IRAGtide. Error bars indicate mean ± SEM of three independent experiments.

In previous studies, we showed that cCMP is able to stimulate purified cAMP- and cGMP-dependent kinases cAK and cGKI [7,8]. Based on these results, we used VASPtide (RRKVSKQE) as a well-established peptide substrate for the determination of cGKI and cAK stimulation. To confirm that cCMP activates kinases and to investigate whether this activation is dependent on the substrate used, we performed (γ-^32^P) ATP kinase assays using purified protein kinases and a novel substrate peptide called IRAGtide (RRRVSVAV) that contains serine 696 as the identified cGKI phosphorylation site of IRAG [22]. Fig 1 demonstrates that both cGKI isoforms (Fig 1A) and cAK (Fig 1B, K_a_(cCMP): 71±11 μM) could be activated by cCMP in a concentration dependent manner. The K_a_(cCMP) for cGKIα and cGKIβ were 26±6 μM and 70±5 μM, respectively. Furthermore, cCMP stimulated the activation of cGKII [K_a_(cCMP): 110±8 μM]. Therefore, both VASPtide and IRAGtide are suitable peptide substrates for the kinase assays. Interestingly, the measured kinase activation differed slightly between the substrate peptides. This result suggests that substrate specificity for these kinases is mediated by peptide sequences not integrated into the phosphorylated consensus sequence.

**Fig 2:**
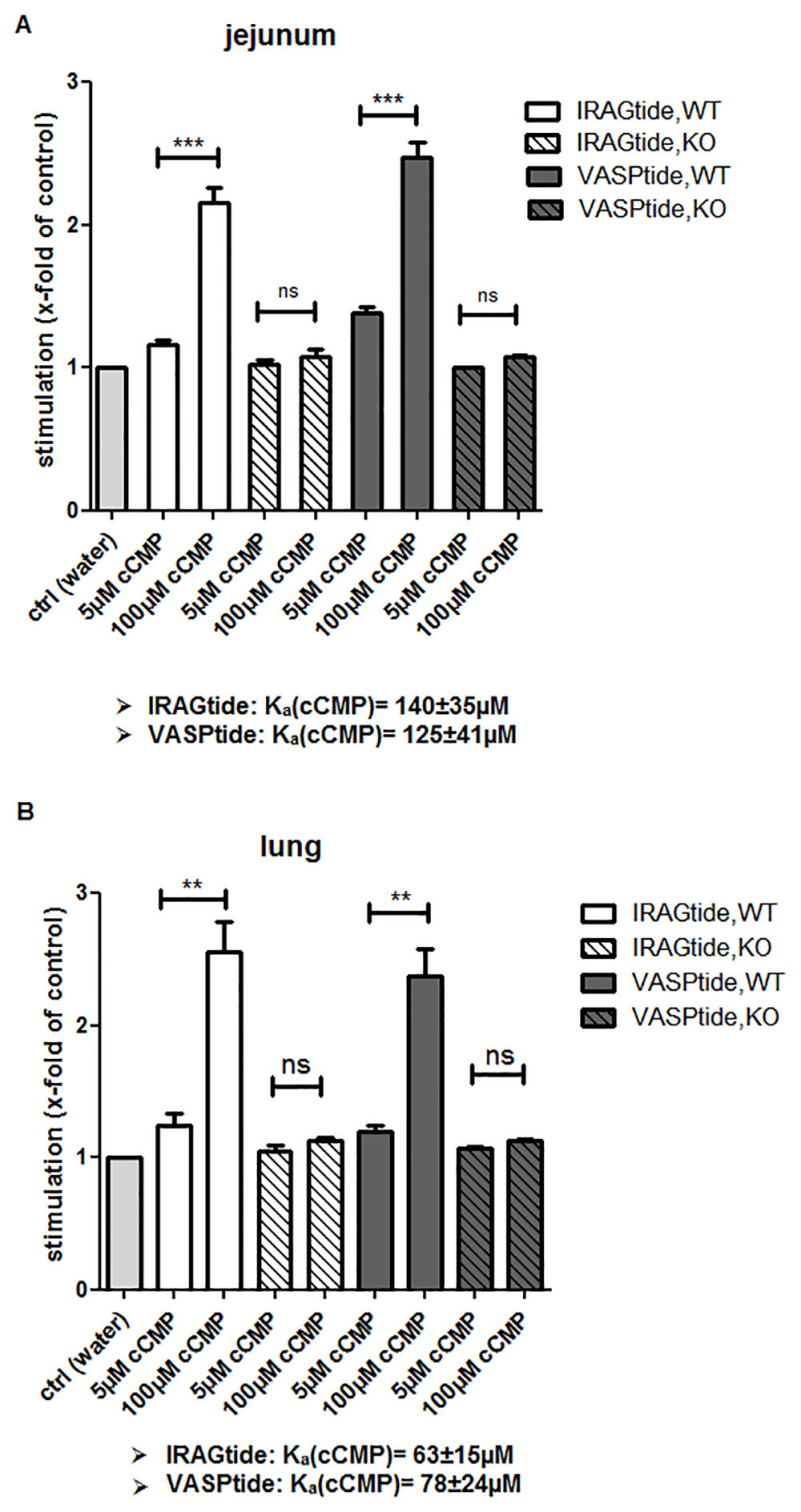
Activation of kinases in tissues of wild type (WT) and cGKI-knockout (KO) mice using two different substrate peptides: VASPtide (RRKVSKQE) and IRAGtide (RRRVSVAV) (A) Stimulation of endogenous cGKs in jejunum tissue lysates after activation with water alone (ctrl) or cCMP (5 or 100 μM) (B) Same panel as (A) using lung tissue lysates. Data were expressed as x-fold stimulation relative to control samples (water alone). Error bars indicate mean ± SEM of four independent experiments. Asterisks indicate statistically significant differences, ns: not statistically significant.

The next step was to examine whether cCMP could stimulate endogenous cGKs in jejunum and lung tissue lysates. Both tissue types are known to contain cGKs in high concentrations [10–12]. Wild-type (WT) and cGKI-knockout (KO) tissue lysates were prepared, and kinase assays were performed as described above. A cAK-inhibitor peptide (AS_5-24_) was added to eliminate cAK phosphorylation. Treatment of WT tissue lysates with cCMP (100 μM) resulted in a 2- to 2.5-fold stimulation compared with water alone (ctrl) (Fig 2). Comparable results were achieved using VASPtide and IRAGtide as the substrate peptides. Interestingly this stimulation of phosphorylation was not detectable in cGKI KO tissue lysates, indicating that cCMP-dependent phosphorylation occurs via cGKI in jejunum and lung tissues. To relate these cCMP mediated effects to cAMP and cGMP we performed (γ-^32^P) ATP kinase assays using increasing cAMP or cGMP concentrations (1 nM-50 mM). S1 Fig shows the activation of kinases at various cNMP concentrations in WT and cGKI KO tissues (lung or jejunum) using the two different substrate peptides. Interestingly the cGMP mediated stimulation of kinases in WT tissue is almost comparable to the cCMP mediated effect, resulting in a 1.8 to 2.8fold stimulation of endogenous kinases (S1A/S1B Fig). The effect of cGMP in WT tissue lysates was completely abolished in cGKI KO tissue lysates as previously observed with cCMP. cAMP enhanced phosphorylation of kinases in WT lung tissue lysate but not in jejunum WT or cGKI KO tissue (S1C/S1D Fig). As a cAK inhibitor peptide (AS_5-24_) was added to eliminate cAK phosphorylation, a crossreaction with other kinases is possible. In these experiments the kinases in jejunum WT tissue possessed a ~70fold lower K_a_ value (2±0.5 μM) for cGMP (S1B Fig) than for cCMP (140±35 μM) (Fig 2A), using IRAGtide as peptide substrate. This fact suggests that higher concentrations of cCMP (relative to cGMP) may be required *in vivo* to activate endogenous kinases. The affinity of cCMP for cGKs is much lower than that of cGMP.

### Identification of cCMP-binding proteins

**Fig 3:**
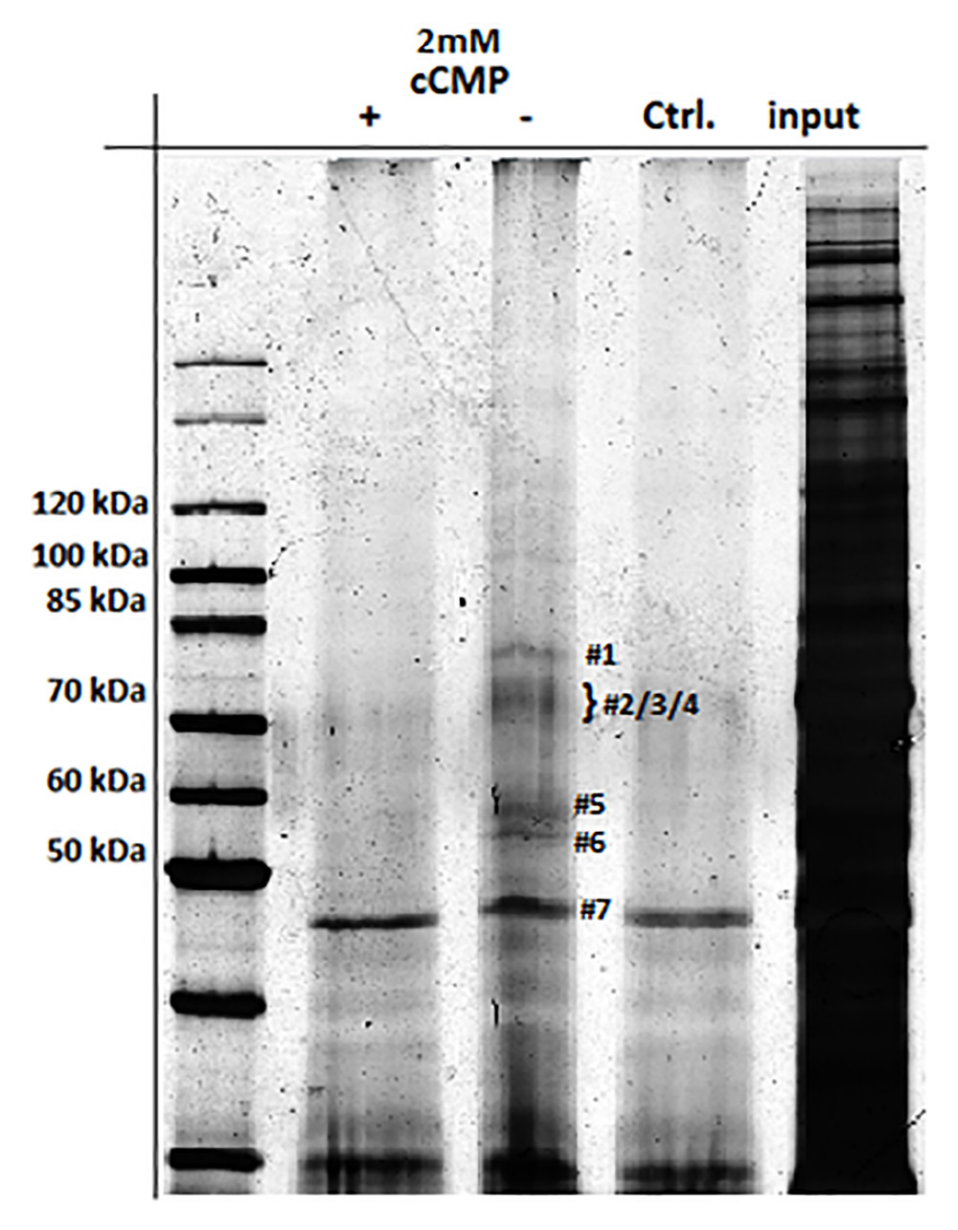
Identification of cCMP-binding proteins. Analysis of lung tissue lysates of wild type mice by immunoprecipitation (using 4-AH-cCMP agarose or EtOH-NH agarose as a control), gel electrophoresis and silver staining. Proteins were detected by mass spectrometric analysis (see S1 Table). cGKI is designated with 1, serum albumin with 2 and 4, HSP7C and 5’-nucleotidase with 3, cAKIIα/Iα reg with 5/6, actin and 60S ribosomal protein L4 with 7.

**Fig 4:**
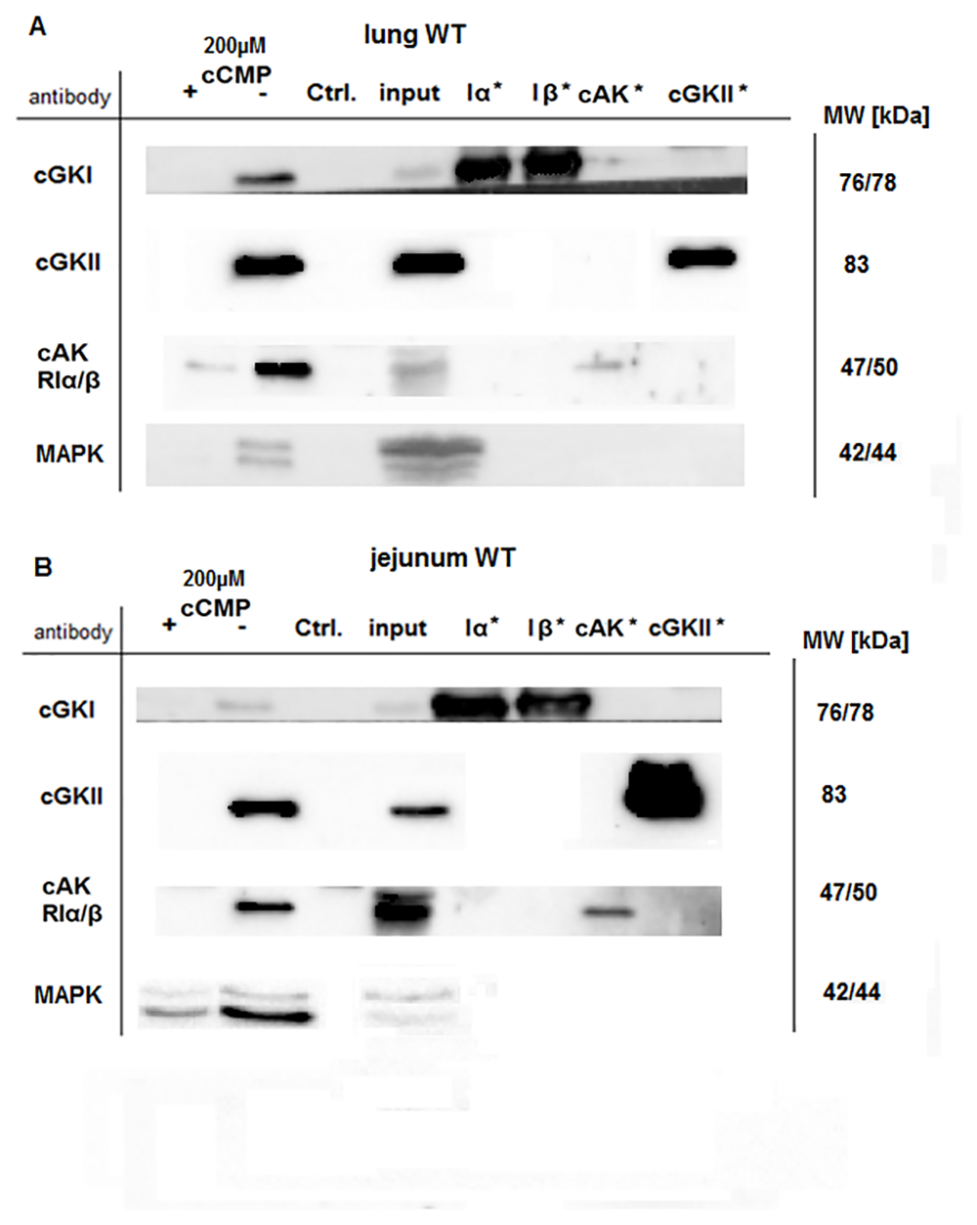
Identification of cCMP-binding proteins (A) Lung tissue lysate (WT) was incubated with 4-AH-cCMP agarose beads or EtOH-NH-agarose beads (ctrl). For the competition experiments, cCMP (200 μM) was added (+) or omitted (-) as described in the Material and Methods. cCMP-binding proteins were analyzed by electrophoresis and immunoblotting with antibodies directed against cGKIc, cGKII, cAKRIα/β or MAPK. Untreated tissue lysate (1 μg/μL, indicated with input) and purified enzyme (1.5 ng/μL) were used as the controls. Purified enzymes are designated with * (B) Same panel as (A) using jejunum (WT) lysate. (S2 Fig) Same experiment depicted in Fig 4 using +/- 2 mM cCMP for competition (WT tissue lysates).

To illuminate the exact role of cCMP, we sought to identify proteins that interact with this cyclic nucleotide. Therefore, lung and jejunum WT tissue lysates were analyzed by cCMP-affinity chromatography and gel electrophoresis of the bound proteins, followed by silver staining (Fig 3, S1 Table) or Western blotting (Fig 4A/4B, S2A/S2B Fig). cCMP was added (+) or omitted (-) during the affinity chromatography experiments to investigate the specificity of the binding. The concentration of 200 μM (Fig 4) or 2 mM cCMP (Fig 3, S2 Fig) was equally used. Using mass spectrometry of silver-stained gel bands, the cGKIα (76 kDa) and cAKIIα (50 kDa)/Iα (47 kDa) reg (regulatory subunits) were identified as cCMP-binding proteins (Fig 3, S1 Table). This binding was verified using Western blotting analysis with the specific primary antibodies cGKIcommon, cGKII and cAKRIα/β, using a more physiological cCMP concentration (200 μM). Fig 4A shows the corresponding immunoblots obtained using lung WT tissue lysate. Interactions between cGKI/cCMP and cGKII/cCMP were also detected in jejunum tissue (Fig 4B). Specific binding of cCMP to cAKRIα/β was also detected. As previously reported [9], we also performed this experiment with 2 mM cCMP (S2A/S2BFig); the effect was the same except that the signals were stronger than with 200 μM cCMP. Furthermore we checked if other cyclic nucleotides, such as cAMP (2 mM) overcome the interaction between cCMP and kinases, such as cGKI, but there was no specific interaction (S2C Fig).Hence, cCMP interacts with both cGK and cAK in the analyzed tissues. Comparable results were obtained using jejunum WT tissue lysate.

### Interaction of MAPK with cGK

**Fig 5:**
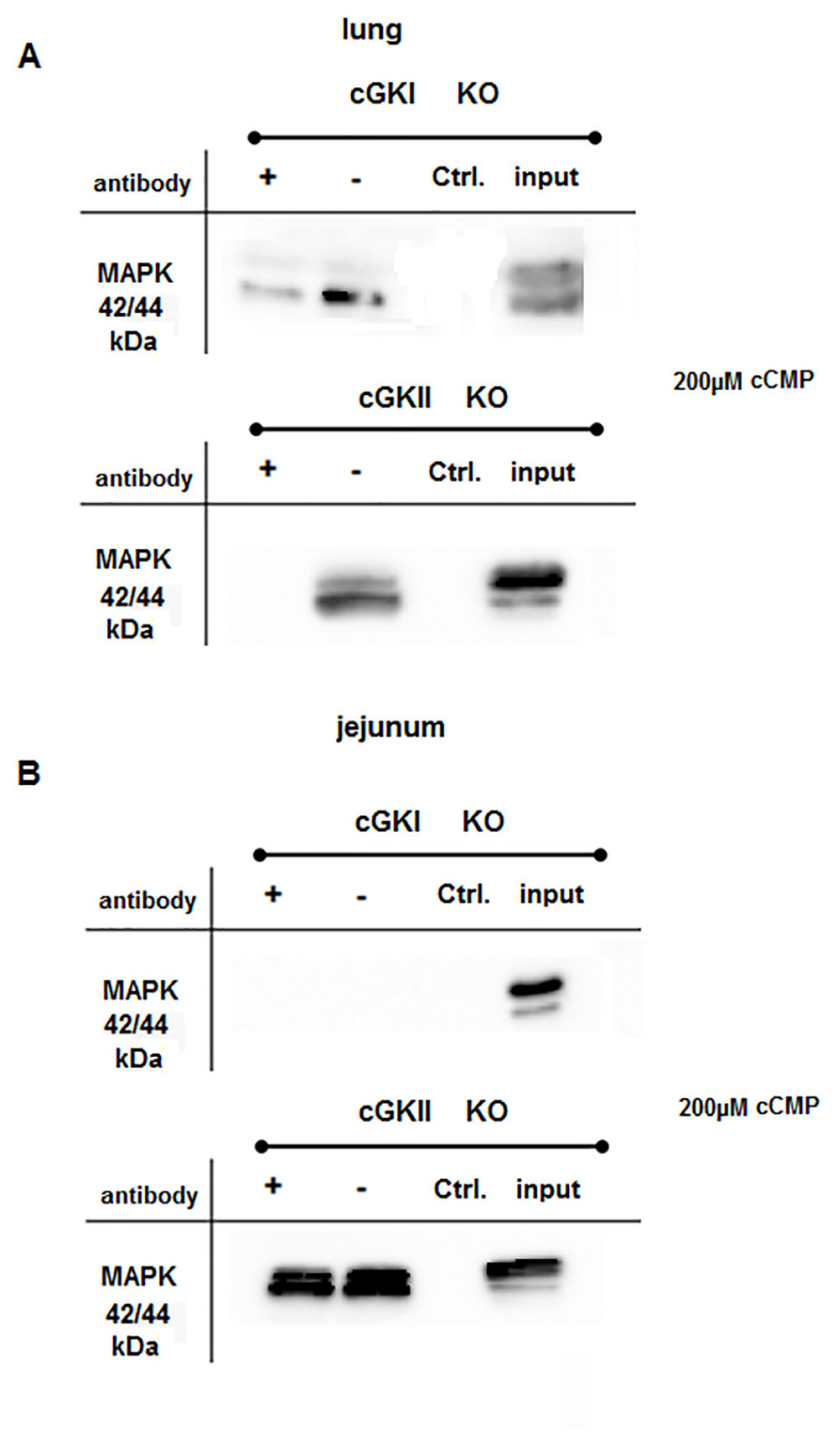
Identification of cCMP-binding proteins (A) Lung tissue lysate (cGKI KO or cGKII KO) was incubated with 4-AH-cCMP agarose beads or EtOH-NH-agarose beads (ctrl). For the competition experiments, cCMP (200 μM) was added (+) or omitted (-) as described in the Material and Methods. Untreated tissue lysate (1 μg/μL, indicated with input) was used as an additional control. cCMP-binding proteins were analyzed by electrophoresis and immunoblotting with an antibody directed against p44/42 MAPK. (B) Same panel as (A) using jejunum tissue lysate. (S3 Fig) Same experiment depicted in Fig 5 using +/- 2 mM cCMP for competition (WT, cGKI KO and cGKII KO tissue lysates).

While searching for other cCMP-interacting proteins, we found several publications that postulated a possible role for cyclic nucleotides and protein kinases interacting with the MAPK (mitogen-activated protein kinase) signaling pathway [23–25]. Therefore, we analyzed whether cCMP interfered with MAPK signaling and detected an interaction between cCMP and p44/42 MAPK. The binding was specific in jejunum (Fig 4B) and lung (Fig 4A) WT tissues, because the addition of an excess of cyclic nucleotide (200 μM cCMP) resulted in the loss of the association of MAPK with the cCMP affinity matrix. Particularly, the p42 MAPK isoform was precipitated in experiments using 200 μM cCMP (Fig 4) or 2 mM cCMP (S2 Fig). To determine whether cGK was essential for this interaction, we performed several competition experiments using tissues from KO mice. MAPK was precipitated from lung cGKI KO and cGKII KO tissues in the same manner as WT tissue (Fig 5A). In contrast, specific binding was only observed using cGKII KO samples in the jejunum tissues (Fig 5B). Working with 2 mM cCMP the same result was achieved (S3 Fig). Interestingly, MAPK was not precipitated from the jejunum cGKI KO tissue lysate (Fig 5B, S3B Fig).

**Fig 6:**
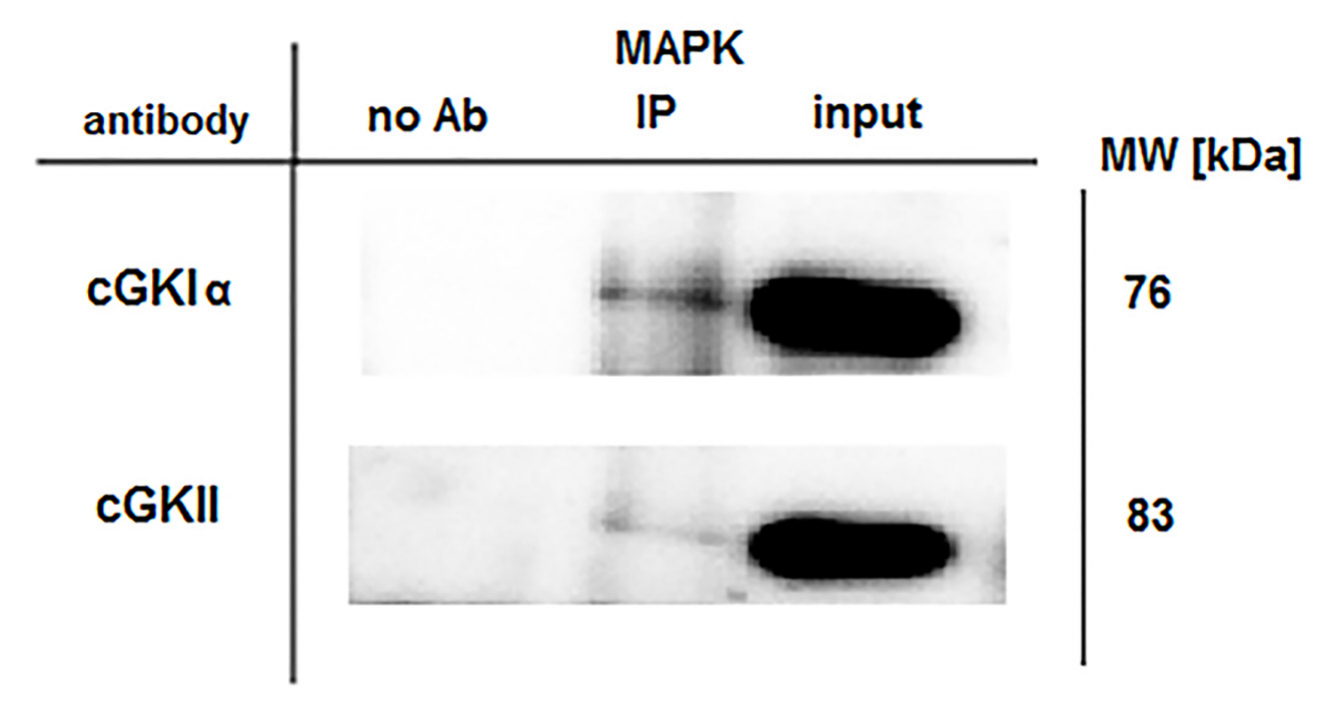
Detection of the protein-protein interaction between cGK and MAPK by co-immunoprecipitation. Jejunum WT tissue lysate was incubated with protein A Sepharose beads coupled with the p44/42 MAPK antibody. After immunoprecipitation, the protein complexes were visualized by Western blotting using antibodies directed against cGKIα and cGKII. For the immunoprecipitation (IP), no antibody (no Ab) was used as a negative control and untreated tissue lysate (1 μg/μL, indicated with input) was used as an additional control.

Next, we performed a co-immunoprecipitation to detect a possible protein-protein interaction between cGK and MAPK (Fig 6). MAPK was precipitated using the cGKIα and cGKII antibodies, indicating an interaction between MAPK, cGK and the cyclic nucleotides. We also performed precipitation studies using 8-AET-cGMP agarose and 200 μM cGMP was added (indicated with +) or omitted (indicated with-). Interestingly, no specific interaction of MAPK with cGMP agarose was detected, both in lung and jejunum WT tissue (S4 Fig).

**Fig 7:**
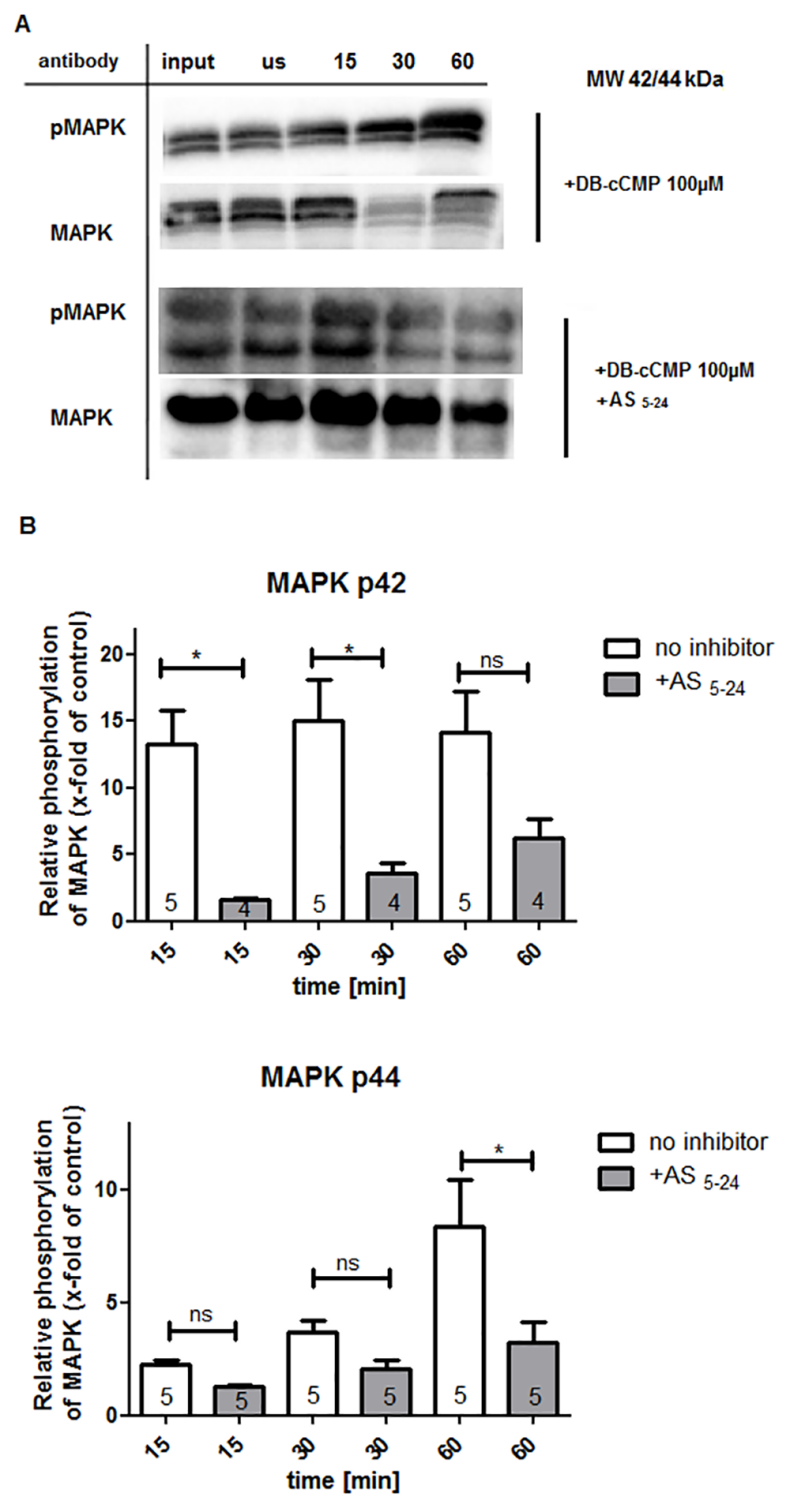
Activation of MAPK by cCMP (A) Jejunum tissue lysate (WT) or lung tissue lysate (WT) (S5 Fig) was treated with 100 μM DB-cCMP for the indicated times (15/30/60 min). A protein kinase A inhibitor (AS _5-24_ cAK inhibitor, 10 μM) was added or omitted (as described in the Material and Methods). As control, untreated (1 μg/μL, indicated with input) and unstimulated tissue lysate (2.5 μg/μL, indicated with ‘us’) was used. Control samples (‘us’) were treated like DB-cCMP stimulated samples but, instead of DB-cCMP, water was added. The phosphorylation of MAPK was detected by immunoblotting using a phospho-p44/42 MAPK antibody (pMAPK). Total MAPK was measured by stripping the membrane and retreating with the respective antibodies. (B) Densitometry analysis of 4–5 independent experiments (numbers in columns) was performed to quantitate p44/42 MAPK levels. Data were expressed as x-fold MAPK phosphorylation relative to untreated control samples. Error bars show mean ± SEM and asterisks indicate statistically significant differences, ns: not statistically significant.

**Fig 8:**
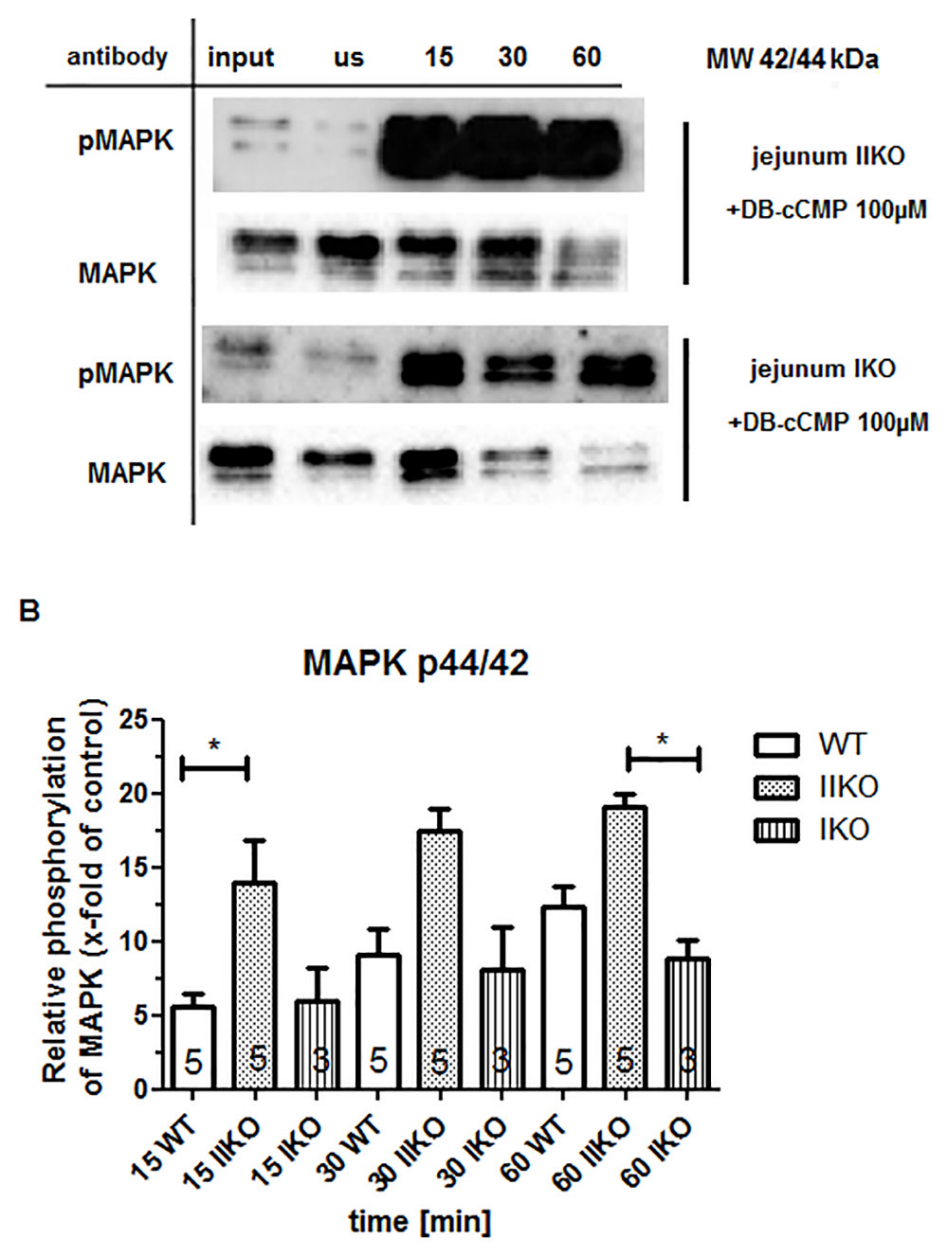
Activation of MAPK by cCMP in (A) cGKII KO tissue or in cGKI KO tissue (both jejunum tissue lysate). Tissue lysate was stimulated with 100 μM DB-cCMP for the indicated times (15/30/60 min). As control, untreated (1 μg/μL, indicated with input) and unstimulated tissue lysate (2.5 μg/μL, indicated with ‘us’) was used. Control samples (‘us’) were treated like DB-cCMP stimulated samples but, instead of DB-cCMP, water was added. The phosphorylation of MAPK was detected by immunoblotting using a pMAPK antibody. Total MAPK was measured by stripping the membrane and retreating with the respective antibody. (B) Densitometry analysis of 3–5 independent experiments (numbers in columns) was performed to quantitate p44/42 MAPK levels. Data were expressed as x-fold MAPK phosphorylation relative to untreated control samples. cGKII KO and cGKI KO data were compared with WT data. Error bars show mean ± SEM. Asterisks indicate statistically significant differences, ns: not statistically significant. The same experiment was conducted with lung tissue lysates (S7 Fig).

Next, we analyzed whether tissue phosphorylation of MAPK was influenced by the addition of the cyclic nucleotide cCMP, and found that the phosphorylation of MAPK was stimulated when 100 μM DB-cCMP was added to the WT tissue lysate for 15–60 min. As a control, an unstimulated tissue lysate was analyzed and compared with the stimulated samples. The control samples (us) were treated identically to the DB-cCMP stimulated samples except that water was added instead of DB-cCMP water. Treatment of lung WT tissue lysate (S5 Fig) with DB-cCMP for 15 min resulted in ~2.5-fold stimulation of p42 MAPK phosphorylation compared to the control, while treatment for 60 min resulted in ~6-fold stimulation. p44 MAPK phosphorylation was less pronounced, but remained significant; the phosphorylation was increased ~2-fold after 15 min of stimulation and ~4-fold after 1 h. The same trend was observed in jejunum WT tissue lysate (Fig 7), with ~13.5-fold stimulation of p42 MAPK phosphorylation after 15 min of stimulation and ~2.5-fold stimulation of p44 MAPK phosphorylation in the same time frame. After stimulation for 60 min, the p44 MAPK phosphorylation level increased to ~8-fold, while the p42 MAPK phosphorylation level stayed the same. In comparison to lung tissue, phosphorylation of MAPK in jejunum tissue was significantly stronger (S6 Fig). In both lung and jejunum tissue, MAPK phosphorylation could be stimulated by cCMP. Interestingly, the phosphorylation of MAPK was inhibited by the addition of the PKA inhibitor AS_5-24_, suggesting a stimulatory function for PKA in cCMP-mediated MAPK phosphorylation. There is a need for further studies analyzing MAPK activation by other cyclic nucleotides. To elucidate the role of cGK in lung and jejunum tissues in this process, we used cGKII knockout (cGKII KO) and cGKI knockout (cGKI KO) mice. The experiment was conducted as described above (the same experiment using WT tissue). We detected phosphorylation both in the jejunum and lung tissues from cGKI KO and cGKII KO mice (Fig 8, S7 Fig). It is interesting to note that the phosphorylation in the jejunum cGKII KO tissue was significantly increased when compared with the WT and cGKI KO tissues; in these tissues, stimulation with 100 μM DB-cCMP for 15 min led to a ~14-fold increase in phosphorylation compared to the unstimulated control, whereas in WT jejunum tissue the increase was only ~6-fold. Interestingly, this effect was not observed in lung tissues. In summary, these results suggest an inhibitory role for cGKII in cCMP-induced MAPK phosphorylation in the jejunum.

## Discussion

In this study, we used cCMP kinase activity assays and affinity chromatography to identify cGKII and MAPK as possible cCMP effector molecules, and confirmed that cGKI and cAK function as cCMP interactors. The activation of the kinase activity of the cGKI isoforms in addition to cGKII and cAK by cCMP using IRAGtide as a substrate is in agreement with previously published data [26]. Interestingly, although the IRAG protein is a specific substrate for cGKIβ, cGKIα phosphorylation was more effective compared to cGKIβ when IRAGtide was used as the substrate. This result indicates that the substrate specificity resulted from an IRAG-cGKIβ interaction site located outside of the kinase domain [22]. Our observations implied that cCMP might modulate diverse tissue functions. The confirmation of the presence of cCMP and cUMP using HPLC and mass spectrometry suggested that the concentrations of these cyclic nucleotides in cells strongly varied [1,27]. Thus, the stimulation of cyclic nucleotide synthesis may diverge in different cells and tissues. Furthermore, effects on different signaling pathways could be exerted in distinct tissues. For example, HCN channels could be modulated by cCMP [28]. In this study, we determined that target proteins of cCMP were present in several tissues, including the lung and jejunum. In addition to the recently established cCMP-binding proteins cGKI and cAKRI, we identified cGKII and MAPK as possible signaling proteins that interact with cCMP. This finding broadens the functions of cCMP. cCMP acts as a signaling molecule; therefore, cCMP might alternatively modulate both the cGK and cAK signaling pathways by acting as a non-canonical second messenger. Interestingly, in previous studies both cCMP and cUMP were detected in the gastric mucosa, indicating a probable function for these molecules in the gastrointestinal tract [29]. The identification of diverse effectors of cCMP could lead to the discovery of its possible roles in the signaling pathways of diverse tissues. For example, cGKI is strongly involved in the relaxation processes. Previously, the role of cCMP via cGKI in vascular relaxation was elucidated using cGKI-knockout mice [7]. Our results investigating the role of cCMP in intestinal and lung tissues may indicate that relaxation in these tissues is *inter alia* regulated by cCMP. The role of cCMP/cGKII in these tissues would most likely be involved in intestinal secretion or lung alveolar fluid clearance [19,30]. As previously discussed [9], cCMP/cAKRI regulates a large variety of cellular processes. However, its role *in vivo* might be less important than that recently described in an investigation into CREB-phosphorylation in S49 lymphoma cells [31]. MAPK stimulation of cAK via Ras/Raf has been demonstrated [32]. Therefore, cCMP may activate MAPK phosphorylation by cAK via a similar signaling pathway. Our results support this stimulatory effect of cAMP/cAK. However, other reports have described the inhibition of MAPK phosphorylation by cAMP/cAK via Rap1 [33]. The newly discovered correlation of cCMP/cGK with MAPK could indicate a role in cellular proliferation. However, this is a highly controversial topic because it is unclear whether cGKI functions in a proliferative or anti-proliferative fashion, and it is not clear how cGKI modulates the signaling of MAPK. Endogenous cGK has been reported to reverse EGF-induced MAPK signaling and thereby inhibit cellular proliferation [34,35]. However, these authors reported that the reduced phosphorylation of p44/42 MAPK by cGMP occurred in a manner that was reversible using a PKG or PKC inhibitor. Additionally, an active role for NO/cGMP/cGKIα on MAPK phosphatase MKP-1 was previously reported in insulin-treated vascular smooth muscle cells [36], and another report showed that p42/p44 MAPK was a potential target of cGKI in pancreatic adenocarcinoma cells, thereby inducing cellular proliferation [25]. There are also divergent results concerning whether MAPK signaling is activated or inhibited by the cGKII isoform. cGKII was shown to inhibit MAPK signaling induced by growth factors, and thereby interfere with the cellular proliferation and migration of breast and gastric cancer cells, respectively [24,37,38]. Our results support a suppressive role for cGKII on MAPK phosphorylation and an inhibitory function on this signaling pathway. cGKII is more strongly expressed in the jejunum than the lung. This finding might explain the reason that this effect was associated with the jejunum tissue lysates. Regardless, the mechanism of action of cGKII is not clear and needs to be elucidated in future studies. It might be possible that MAPK phosphatases are involved or directly targeted by cGKII. However, other reports have suggested that intestinal cell proliferation might be stimulated by guanylyl cyclase via cGKII/MAPK [23]. Here, we showed that a complex of cGKI/MAPK or cGKII/MAPK may be activated by cCMP. However, how this complex is formed and whether adaptor proteins are necessary to assemble this kinase complex is unknown. It would be interesting to discover whether these complexes regulate cellular processes in diverse tissues. Further studies are necessary to elucidate these mechanisms and to establish the probable cellular functions of cCMP effector proteins.

## Supporting Information

S1 Fig Activation of kinases in tissues of wild type (WT) and cGKI-knockout (KO) mice using two different substrate peptides: VASPtide (RRKVSKQE) and IRAGtide (RRRVSVAV). (A/B) Stimulation of endogenous cGKs in lung or jejunum tissue lysates after activation with water alone (ctrl) or cGMP (50 nM or 5 μM). (C/D) Same panel as (A/B) using cAMP (500 nM or 100 μM). Data were expressed as x-fold stimulation relative to control samples (water alone). Error bars indicate mean±SEM of three independent experiments. Asterisks indicate statistically significant differences, ns: not statistically significant.
(TIF)

S2 Fig Identification of cCMP-binding proteins (A) Lung tissue lysate (WT) was incubated with 4-AH-cCMP agarose beads or EtOH-NH-agarose beads (ctrl). For the competition experiments, cCMP (2 mM) was added (+) or omitted (-) as described in the Material and Methods. Untreated tissue lysate (1 μg/μL, indicated with input) and purified enzyme (1.5 ng/μL) were used as the controls. Purified enzymes are designated with * (B) Same panel as (A) using jejunum (WT) lysate. cCMP-binding proteins were analyzed by electrophoresis and immunoblotting with antibodies directed against cGKIc, cGKII or cAKRIα/β (C) Jejunum WT tissue lysate was incubated with 4-AH-cCMP agarose beads or EtOH-NH-agarose beads (ctrl). For the competition experiments, cAMP (2 mM) was added (+) or omitted (-). Untreated tissue lysate (1 μg/μL, indicated with input) was used as control; bound proteins were analyzed by electrophoresis and immunoblotting (cGKIc antibody).
(TIF)

S3 Fig Identification of cCMP-binding proteins (A) Lung tissue lysate (WT, cGKI KO and cGKII KO) was incubated with 4-AH-cCMP agarose beads or EtOH-NH-agarose beads (ctrl). For the competition experiments, cCMP (2 mM) was added (+) or omitted (-) as described in the Material and Methods. Untreated tissue lysate (1 μg/μL, indicated with input) was used as an additional control. cCMP-binding proteins were analyzed by electrophoresis and immunoblotting with an antibody directed against p44/42 MAPK (B) Same panel as (A) using jejunum tissue lysate (WT, cGKI KO and cGKII KO).
(TIF)

S4 Fig Identification of cGMP-binding proteins. Lung and jejunum WT tissue lysates were incubated with 8-AET-cGMP agarose beads or EtOH-NH-agarose beads (ctrl). For the competition experiments, cGMP (200 μM) was added (+) or omitted (-) as described in the Material and Methods. Untreated tissue lysate (1 μg/μL, indicated with input) was used as an additional control. cGMP-binding proteins were analyzed by electrophoresis and immunoblotting with antibodies directed against cGKIc, cGKII or p44/42 MAPK.
(TIF)

S5 Fig Activation of MAPK by cCMP. Lung tissue lysate (WT) was treated with 100 μM DB-cCMP for the indicated times (15/30/60 min). A protein kinase A inhibitor (AS _5-24_ cAK inhibitor, 10 μM) was added or omitted (as described in the Material and Methods). The phosphorylation of MAPK was detected by immunoblotting using a phospho-p44/42 MAPK antibody (pMAPK). Total MAPK was measured by stripping the membrane and retreating with the respective antibodies. Densitometry analysis of 4–6 independent experiments (numbers in columns) was performed to quantitate the p44/42 MAPK levels. Data were expressed as x-fold MAPK phosphorylation relative to untreated control samples. Error bars show mean ± SEM. Asterisks indicate statistically significant differences, ns: not statistically significant.
(TIF)

S6 Fig Activation of MAPK by cCMP. WT tissue lysates (lung or jejunum) were treated with 100 μM DB-cCMP for the indicated times (15/30/60 min). The phosphorylation of MAPK was detected by immunoblotting using a phospho-p44/42 MAPK antibody (pMAPK). Total MAPK was measured by stripping the membrane and retreating with the respective antibodies. Densitometry analysis of 5–6 independent experiments (numbers in columns) was performed to quantitate pMAPK levels. Data were expressed as x-fold MAPK phosphorylation relative to untreated control samples. Error bars show mean ± SEM. Asterisks indicate statistically significant differences, ns: not statistically significant.
(TIF)

S7 Fig Activation of MAPK by cCMP in cGKII KO tissue or in cGKI KO tissue (both lung tissue lysate). Tissue lysate was stimulated with 100 μM DB-cCMP for the indicated times (15/30/60 min). As control, untreated (1 μg/μL, indicated with input) and unstimulated tissue lysate (2.5 μg/μL, indicated with ‘us’) was used. Control samples (‘us’) were treated like DB-cCMP stimulated samples but, instead of DB-cCMP, water was added. The phosphorylation of MAPK was detected by immunoblotting using pMAPK antibody. Total MAPK was measured by stripping the membrane and retreating with the respective antibody. Densitometry analysis of 3–6 independent experiments (numbers in columns) was performed to quantitate p44/42 MAPK levels. Data were expressed as x-fold MAPK phosphorylation relative to untreated control samples. cGKII KO and cGKI KO data were compared with WT data. Error bars show mean ± SEM. Asterisks indicate statistically significant differences, ns: not statistically significant.
(TIF)

S1 Table Identification of cCMP-binding proteins in lung WT tissue lysate via cCMP agarose precipitation, gel electrophoresis, silver staining (Fig 3) and mass spectrometric analysis. Bands of interest were excised from the silver stained gel and handled as described in Material and Methods. The analysis was carried out using a nano-LC-MS/MS system and the tandem MS-spectra were aligned with the Uniprot-database.
(DOCX)
