# Supplementary figures and images for: Interaction of cCMP with the cGK, cAK and MAPK Kinases in Murine Tissues
Source: PLoS One. 2015 May 15;10(5):e0126057. doi: 10.1371/journal.pone.0126057 (PMC4433244; doi:10.1371/journal.pone.0126057)

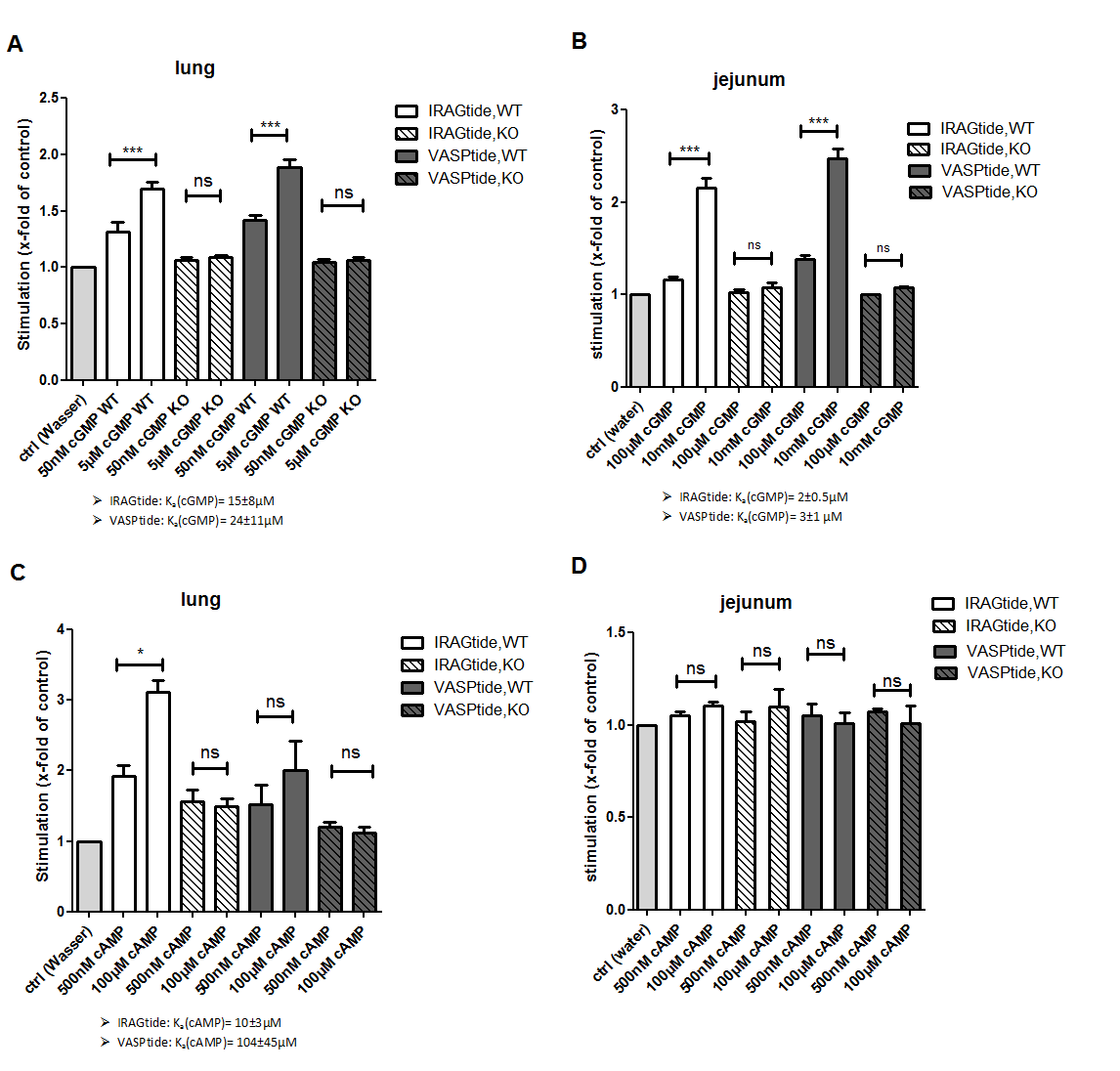

Supplement: S1 Fig — (A/B) Stimulation of endogenous cGKs in lung or jejunum tissue lysates after activation with water alone (ctrl) or cGMP (50 nM or 5 μM). (C/D) Same panel as (A/B) using cAMP (500 nM or 100 μM). Data were expressed as x-fold stimulation relative to control samples (water alone). Error bars indicate mean±SEM of three independent experiments. Asterisks indicate statistically significant differences, ns: not statistically significant. (TIF) [file pone.0126057.s001.tif]

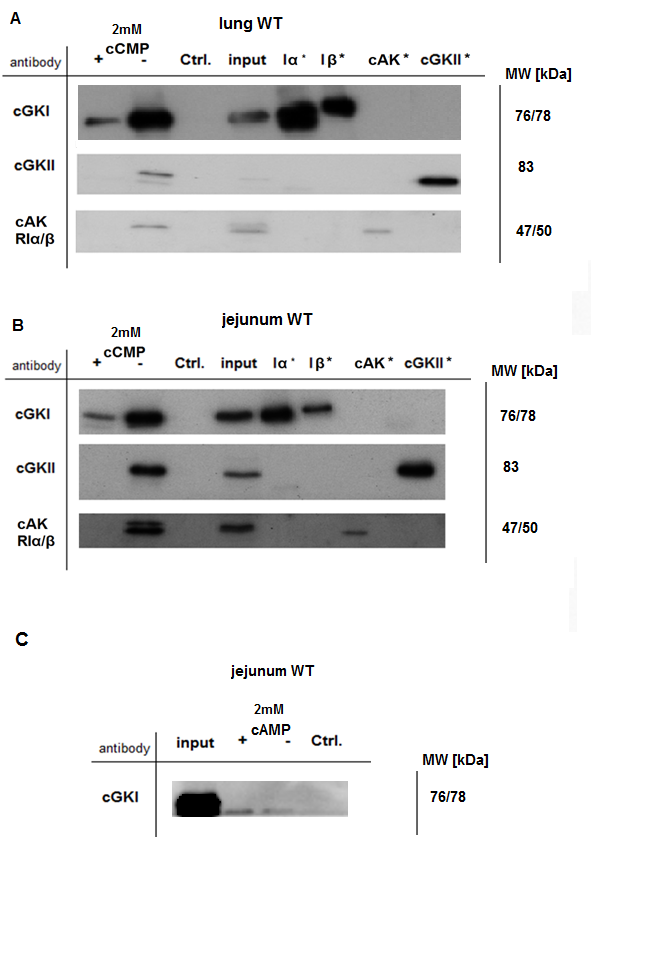

Supplement: S2 Fig — For the competition experiments, cCMP (2 mM) was added (+) or omitted (-) as described in the Material and Methods. Untreated tissue lysate (1 μg/μL, indicated with input) and purified enzyme (1.5 ng/μL) were used as the controls. Purified enzymes are designated with * (B) Same panel as (A) using jejunum (WT) lysate. cCMP-binding proteins were analyzed by electrophoresis and immunoblotting with antibodies directed against cGKIc, cGKII or cAKRIα/β (C) Jejunum WT tissue lysate was incubated with 4-AH-cCMP agarose beads or EtOH-NH-agarose beads (ctrl). For the competition experiments, cAMP (2 mM) was added (+) or omitted (-). Untreated tissue lysate (1 μg/μL, indicated with input) was used as control; bound proteins were analyzed by electrophoresis and immunoblotting (cGKIc antibody). (TIF) [file pone.0126057.s002.tif]

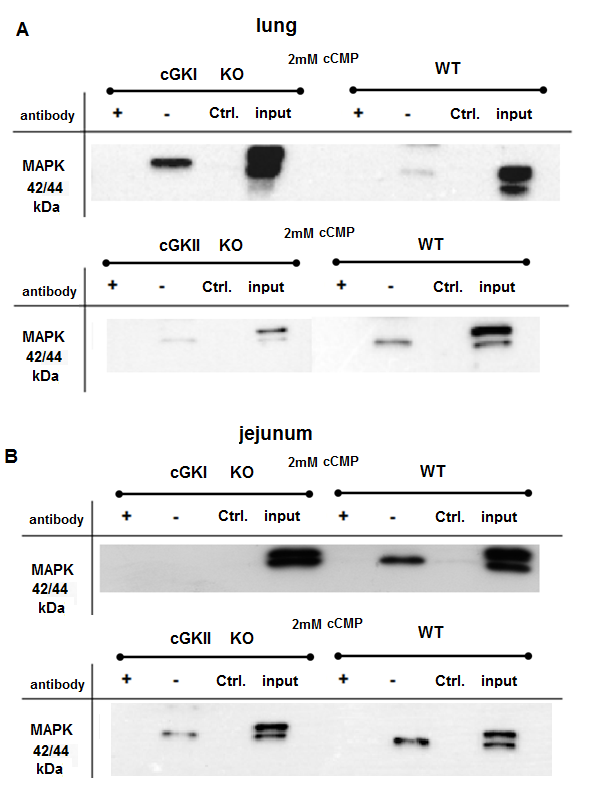

Supplement: S3 Fig — For the competition experiments, cCMP (2 mM) was added (+) or omitted (-) as described in the Material and Methods. Untreated tissue lysate (1 μg/μL, indicated with input) was used as an additional control. cCMP-binding proteins were analyzed by electrophoresis and immunoblotting with an antibody directed against p44/42 MAPK (B) Same panel as (A) using jejunum tissue lysate (WT, cGKI KO and cGKII KO). (TIF) [file pone.0126057.s003.tif]

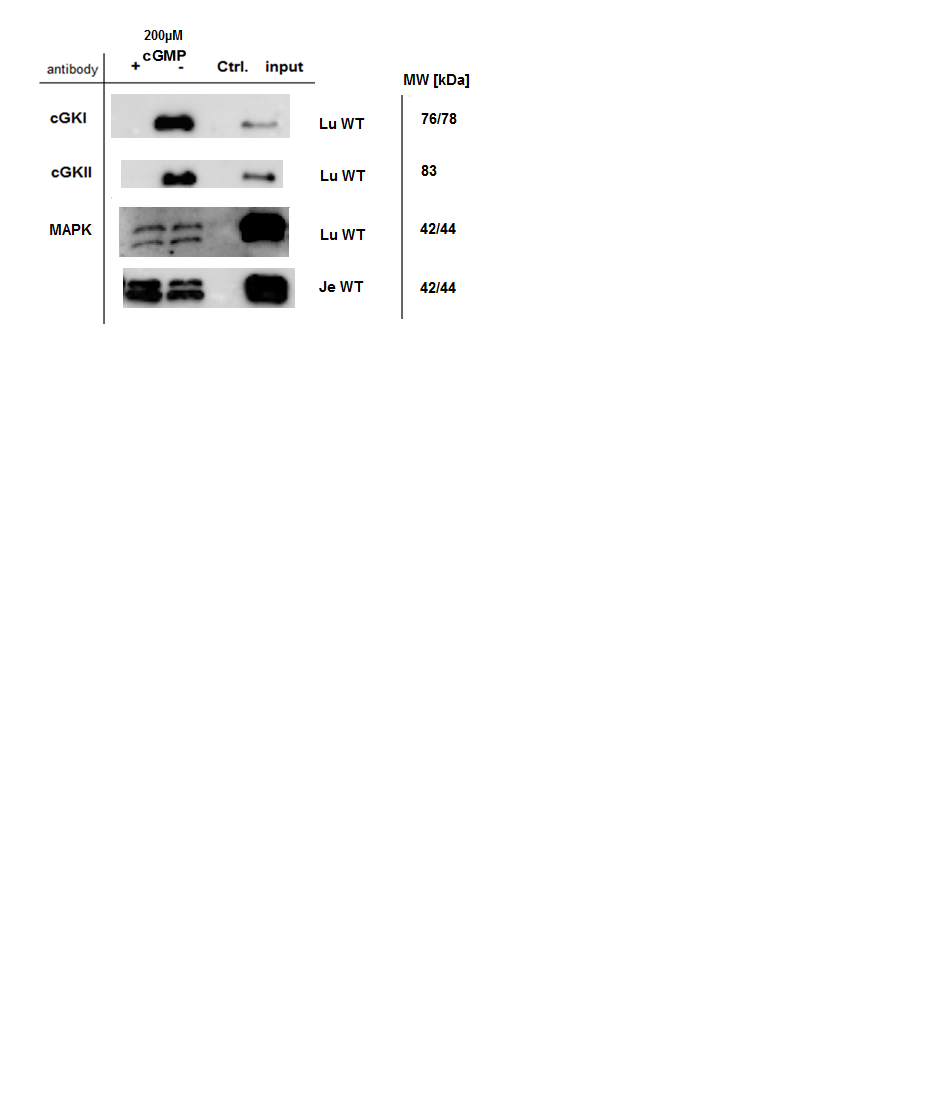

Supplement: S4 Fig — Lung and jejunum WT tissue lysates were incubated with 8-AET-cGMP agarose beads or EtOH-NH-agarose beads (ctrl). For the competition experiments, cGMP (200 μM) was added (+) or omitted (-) as described in the Material and Methods. Untreated tissue lysate (1 μg/μL, indicated with input) was used as an additional control. cGMP-binding proteins were analyzed by electrophoresis and immunoblotting with antibodies directed against cGKIc, cGKII or p44/42 MAPK. (TIF) [file pone.0126057.s004.tif]

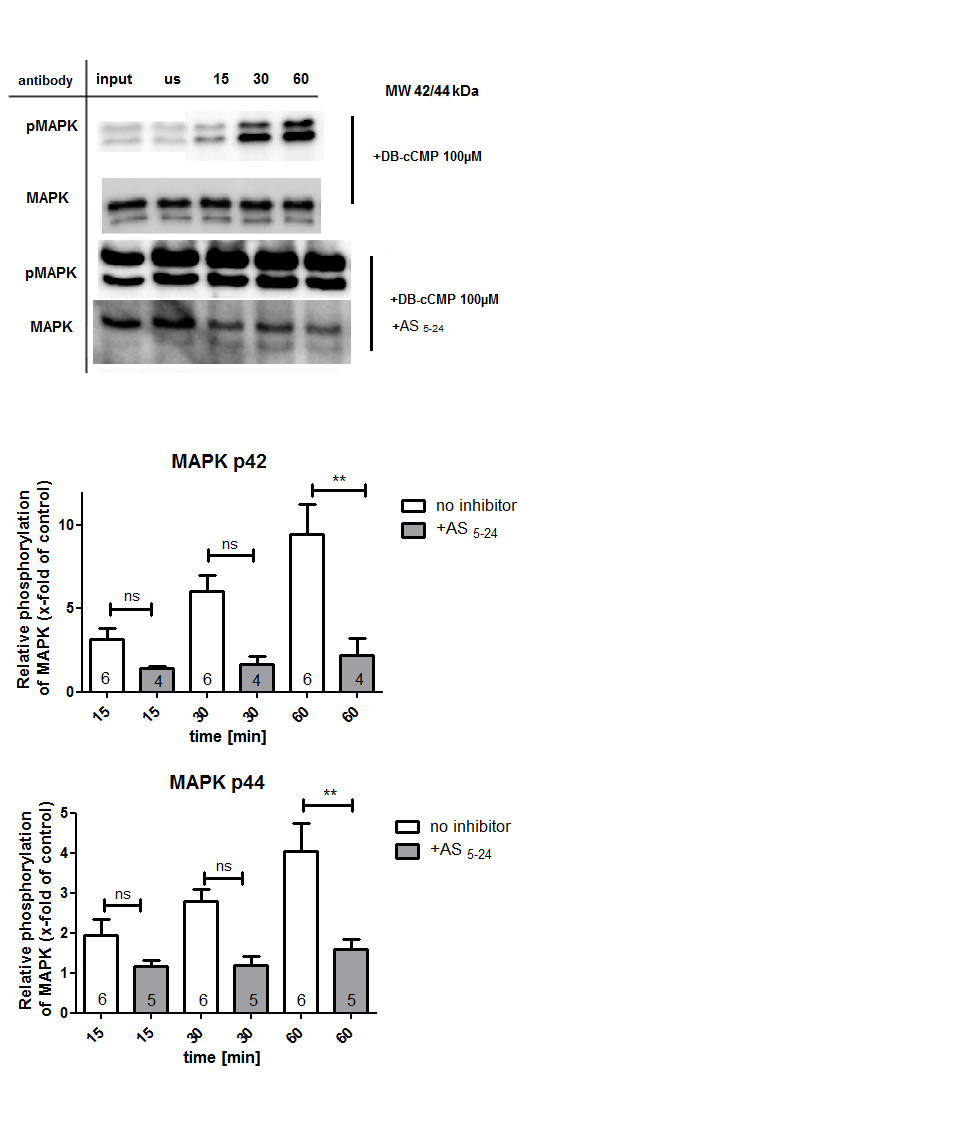

Supplement: S5 Fig — Lung tissue lysate (WT) was treated with 100 μM DB-cCMP for the indicated times (15/30/60 min). A protein kinase A inhibitor (AS 5-24 cAK inhibitor, 10 μM) was added or omitted (as described in the Material and Methods). The phosphorylation of MAPK was detected by immunoblotting using a phospho-p44/42 MAPK antibody (pMAPK). Total MAPK was measured by stripping the membrane and retreating with the respective antibodies. Densitometry analysis of 4–6 independent experiments (numbers in columns) was performed to quantitate the p44/42 MAPK levels. Data were expressed as x-fold MAPK phosphorylation relative to untreated control samples. Error bars show mean ± SEM. Asterisks indicate statistically significant differences, ns: not statistically significant. (TIF) [file pone.0126057.s005.tif]

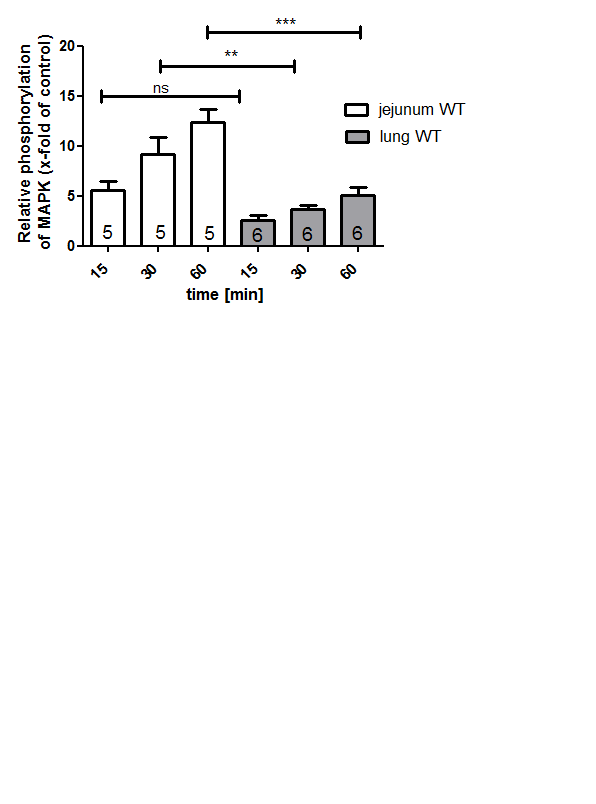

Supplement: S6 Fig — WT tissue lysates (lung or jejunum) were treated with 100 μM DB-cCMP for the indicated times (15/30/60 min). The phosphorylation of MAPK was detected by immunoblotting using a phospho-p44/42 MAPK antibody (pMAPK). Total MAPK was measured by stripping the membrane and retreating with the respective antibodies. Densitometry analysis of 5–6 independent experiments (numbers in columns) was performed to quantitate pMAPK levels. Data were expressed as x-fold MAPK phosphorylation relative to untreated control samples. Error bars show mean ± SEM. Asterisks indicate statistically significant differences, ns: not statistically significant. (TIF) [file pone.0126057.s006.tif]

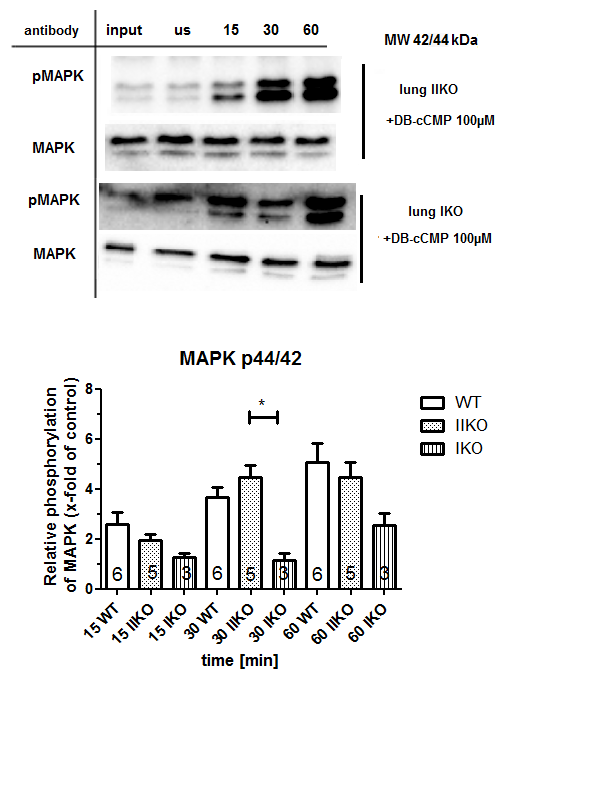

Supplement: S7 Fig — Tissue lysate was stimulated with 100 μM DB-cCMP for the indicated times (15/30/60 min). As control, untreated (1 μg/μL, indicated with input) and unstimulated tissue lysate (2.5 μg/μL, indicated with ‘us’) was used. Control samples (‘us’) were treated like DB-cCMP stimulated samples but, instead of DB-cCMP, water was added. The phosphorylation of MAPK was detected by immunoblotting using pMAPK antibody. Total MAPK was measured by stripping the membrane and retreating with the respective antibody. Densitometry analysis of 3–6 independent experiments (numbers in columns) was performed to quantitate p44/42 MAPK levels. Data were expressed as x-fold MAPK phosphorylation relative to untreated control samples. cGKII KO and cGKI KO data were compared with WT data. Error bars show mean ± SEM. Asterisks indicate statistically significant differences, ns: not statistically significant. (TIF) [file pone.0126057.s007.tif]
