# Supplementary material for: Interaction of cCMP with the cGK, cAK and MAPK Kinases in Murine Tissues
Source: PLoS One. 2015 May 15;10(5):e0126057. doi: 10.1371/journal.pone.0126057 (PMC4433244; doi:10.1371/journal.pone.0126057)
Supplement: S1 Table — Bands of interest were excised from the silver stained gel and handled as described in Material and Methods. The analysis was carried out using a nano-LC-MS/MS system and the tandem MS-spectra were aligned with the Uniprot-database. (DOCX) [file pone.0126057.s008.docx]

Table S1. Identification of cCMP-binding proteins via immunoprecipitation, gel electrophoresis, silver staining and mass spectrometric analysis

| Band | Sequence Coverage [%] | Accession | Name | Species | Peptides(95%) | Peptide Confidence [%] | Peptide Sequence |
| --- | --- | --- | --- | --- | --- | --- | --- |
| 1 | 8,2 | P0C605 | cGMP-dependent protein kinase 1 | MOUSE | 1 | 99 | EDSPSEDPVFLR |
|  |  |  |  |  |  | 77,4 | GDTFFIISK |
|  |  |  |  |  |  | 64,2 | GIDM(ox)IEFPK |
| 2 | 3,6 | P02769 | Serum albumin | BOVIN | 2 | 99 | LVNELTEFAK |
|  |  |  |  |  |  | 99 | TVM(ox)ENFVAFVDK |
| 3 | 27,2 | P02769 | Serum albumin | BOVIN | 9 | 99 | DDPHACYSTVFDK |
|  |  |  |  |  |  | 99 | KVPQVSTPTLVEVSR |
|  |  |  |  |  |  | 99 | LGEYGFQNALIVR |
|  |  |  |  |  |  | 99 | LVNELTEFAK |
|  |  |  |  |  |  | 99 | TVM(ox)ENFVAFVDK |
|  |  |  |  |  |  | 98,6 | YICDNQDTISSK |
|  |  |  |  |  |  | 98,5 | CCTESLVNR |
|  |  |  |  |  |  | 97,8 | EACFAVEGPK |
|  |  |  |  |  |  | 91 | CCAADDKEACFAVEGPK |
|  |  |  |  |  |  | 80,4 | AEFVEVTK |
|  | 12,9 | P63017 | Heat shock cognate 71 kDa protein | MOUSE | 6 | 99 | DAGTIAGLNVLR |
|  |  |  |  |  |  | 99 | IINEPTAAAIAYGLDK |
|  |  |  |  |  |  | 99 | VEIIANDQGNR |
|  |  |  |  |  |  | 97,7 | STAGDTHLGGEDFDNR |
|  |  |  |  |  |  | 95 | TTPSYVAFTDTER |
|  |  |  |  |  |  | 98,2 | NQVAM(ox)NPTNTVFDAK |
|  | 3,5 | Q61503 | 5'-nucleotidase | MOUSE | 2 | 99 | VVYPAVEGR |
|  |  |  |  |  |  | 99 | YPFIVTADDGR |
| 4 | 8,9 | P02769 | Serum albumin | BOVIN | 2 | 99 | LVNELTEFAK |
|  |  |  |  |  |  | 99 | YICDNQDTISSK |
|  |  |  |  |  |  | 85,7 | EYEATLEECCAK |
|  |  |  |  |  |  | 89 | TVM(ox)ENFVAFVDK |
| 5 | 26,2 | P12367 | cAMP-dependent protein kinase type II-alpha regulatory subunit | MOUSE | 3 | 99 | AATIIATSEGSLWGLDR |
|  |  |  |  |  |  | 96,6 | GTYDILVTK |
|  |  |  |  |  |  | 95 | CLVMDVQAFER |
|  |  |  |  |  |  | 84,5 | IVDVIGEK |
|  |  |  |  |  |  | 80,1 | GSFGELALM(ox)YNTPR |
| 6 | 27,3 | Q9DBC7 | cAMP-dependent protein kinase type I-alpha regulatory subunit | MOUSE | 1 | 99 | SENEEFVEVGR |
|  |  |  |  |  |  | 94,1 | LTVADALEPVQFEDGQK |
|  |  |  |  |  |  | 93,8 | GAISAEVYTEEDAASYVR |
|  |  |  |  |  |  | 76,2 | VSILESLDK |
|  |  |  |  |  |  | 50 | VLGPCSDILK |
| 7 | 40 | P63260 | Actin, cytoplasmic 2 | MOUSE | 8 | 99 | AGFAGDDAPR |
|  |  |  |  |  |  | 99 | EITALAPSTMK |
|  |  |  |  |  |  | 99 | GYSFTTTAER |
|  |  |  |  |  |  | 99 | (p)QEYDESGPSIVHR |
|  |  |  |  |  |  | 99 | SYELPDGQVITIGNER |
|  |  |  |  |  |  | 99 | VAPEEHPVLLTEAPLNPK |
|  |  |  |  |  |  | 99 | DLYANTVLSGGTTM(ox)YPGIADR |
|  |  |  |  |  |  | 71,8 | AVFPSIVGR |
|  |  |  |  |  |  | 64,1 | LCYVALDFEQEMATAASSSSLEK |
|  | 27,2 | Q9D8E6 | 60S ribosomal protein L4 | MOUSE | 6 | 99 | GPCIIYNEDN(deam)GIIK |
|  |  |  |  |  |  | 99 | IEEVPELPLVVEDK |
|  |  |  |  |  |  | 99 | NIPGITLLNVSK |
|  |  |  |  |  |  | 99 | (p)QPYAVSELAGHQTSAESWGTGR |
|  |  |  |  |  |  | 98,9 | MMNTDLSR |
|  |  |  |  |  |  | 96,2 | SGQGAFGNMCR |
|  |  |  |  |  |  | 73,3 | NVTLPAVFK |
